# Diseases of the Stomach and Intestines

**Published:** 1886-11

**Authors:** 


**Diseases of the Stomach and Intestines.** A Manual of Clinical Therapeutics, for the student and Practitioner. By PROF. DUJARDIN-BEAUMETZ, Physician to the Cochin Hospital, etc., etc. Translated from the fourth French edition by E. P. Hurd, M. D.; with illustrations and one chromo lithograph. New York: Wm Wood & Co.

The late issues of "Wood's Library" have been exceptionally valuable. Those of our readers who are not subscribers we would urge to send in their names. They cannot make a better investment of the same amount of money. This volume of Prof. Dujardin-Beaumetz is devoted especially to the *Treatment* of diseases of the stomach and intestines, and recognizing that the administration of drugs is secondary; he devotes much attention to food and alimentation in these diseases. The book is full of valuable instruction, which cannot be otherwise had in the present compact and easily referred to form.

